# Social support moderates the association of functional difficulty with major depression among community-dwelling older adults: evidence from LASI, 2017–18

**DOI:** 10.1186/s12888-022-03959-3

**Published:** 2022-05-04

**Authors:** T. Muhammad, Priya Maurya

**Affiliations:** 1 Department of Family and Generations, International Institute for Population Sciences, Deonar, Mumbai, Maharashtra India 400088; 2 Department of Population and Development, International Institute for Population Sciences, Mumbai, Maharashtra India 400088

**Keywords:** Social support, Functional ability, Major depressive disorder, Older adults

## Abstract

**Background:**

This study aimed to examine the potential independent association of functional disability with major depression and moderating effects of social support variables including marital status, living arrangement and social participation in such associations.

**Methods:**

Data for the study were drawn from the Longitudinal Ageing Study in India (LASI) wave 1 that was collected during 2017–18 including a sample of 31,464 individuals aged 60 years and above. Descriptive statistics and results from bivariate analysis have been reported. Further, moderated multivariable logistic regression models were used to fulfil the study objective. Major depressive disorder was assessed using the scale of the Short Form Composite International Diagnostic Interview (CIDI-SF).

**Results:**

It was found that 8.67% of older participants were depressed in this study. Older adults who had difficulty in basic activities of daily living (BADL) (15.34%), difficulty in instrumental activities of daily living (IADL) (12.06%), unmarried (10.13%), separate living (9.67%) and socially inactive (10.09) were having higher prevalence of major depression compared to their respective counterparts. The adjusted model-1 revealed that older adults who had difficulty in BADL and IADL were 2.53 times [AOR: 2.53, CI: 2.17—2.95] and 2.27 times [AOR: 2.27, CI: 1.97—2.64] more likely to have major depression than those with no difficulty in BADL and IADL respectively. Further, interaction analyses found that currently unmarried status, separate living and being socially inactive have moderation effects in the observed associations and exacerbate the likelihood of major depression among older adults who are functionally impaired.

**Conclusions:**

The findings highlight the importance of integrating social participation in the daily life of older adults and developing initiatives that promote a healthy surrounding such as social connectedness, co-residential living and special care for those who are physically disabled to protect against late-life depression.

## Background

India has the second largest proportion of older population in the world after China. The expanding share of aging population in the country concerns around the increasingly rising need for developing long-term care services and ensuring physical and mental well-being in adults in their later life [1]. Since the physical and functional abilities tend to decline by increasing age, absence of care and support sources may exacerbate the illbeing of older adults and may develop mental disorders in old age [2–4]. Depression is the major mental health problem and leading cause of disability worldwide [5]. A pooled analysis of fifty-one studies from India reported that 34.4% of elderly population are suffuring from depression [6].

Multiple studies in high, middle and low-income countries have shown that functional disability, quality of life, and chronic conditions were strongly associated with depressive symptoms in the past 12 months [7–10]. Functional disability has consistently been identified as a crucial stressor contributing to poor well-being and depression [11–15]. Similarly, research has found that measures of physical functioning especially the activities of daily living (ADL) or instrumental activities of daily living (IADL) are related to feelings of well-being and any failure to carry out those has been found to trigger mental illnesses among older people [16–19]. It is also observed that older people’s inability to do their daily activities may create feelings of dependence and undermine their autonomy, which in turn lead to compromised daily mood and heightened depressive symptoms [20, 21].

Social support is generally defined as individuals’ relationships with other people including formal and informal ones, such as family, relatives, friends, peers, or community organizations [22], having an association with improved wellbeing. In Indian context, family is an institution which lays the seeds of social cohesion and provides social security and economic support to the older population. Also, residing with family is most preferred and satisfactory living arrangement among older people in India which is associated with improved psychological wellbeing [23–25]. A recent study in India found that older adults who lived alone and were widowed had higher chances of depression [26]. Similarly, the impaired social relationships are associated with depression, and other mental and physical illnesses [27]. Importantly, any disruptions in familial relationships may have greater negative impact on well-being than conflicts in other relationships, as they threaten enduring commitments [28]. On the other hand, spousal loss is considered one of life’s most stressful experiences and its detrimental consequences on mental health have been documented in numerous cross-sectional as well as longitudinal studies [29–31]. Similarly, living arrangement can be a structural factor of social support and a measure of real-life social bonds [32, 33], and is one of the well-established risk factors for depressive symptoms among older adults [34, 35]. In the context of age-related impairments of solo-living older people, their health behaviours and decisions may become critical which may result in further functional decline and increased depressive symptoms [36–38]. Thus, difficulties in physical functioning can be detrimental to mental wellbeing depending on the level of available structural and social support. A growing body of literature report that participation in various kinds of social activities such as eating out, visiting friends and relatives, and swimming was associated with better psychological well-being and less depressive symptoms among older adults. It has also been found that participation in such social activities and group exercises reduces depressive symptoms among older adults who are functionally impaired [39, 40]. Furthermore, another study found that participation in leisure activities also significantly buffered the relationship between functional difficulty and depression in older individuals [41].

**Fig. 1:**
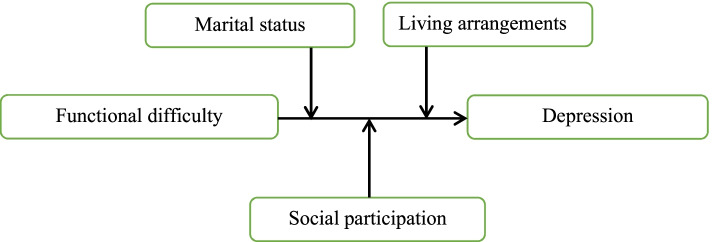
Conceptual framework for the study

However, little is known about how different sources of social support may influence the associations between functional disability and mental disorders among older people in India. Examining the moderating role of specific indicators of social support in these associations may be informative for future targeted interventions. Hence, in this study, we examined the potential independent association of functional disability with depression and moderating effects of social support variables including marital status, living arrangement and social participation in such associations (Fig. 1), hypothesizing that widowhood, living alone and lack of social participation would exacerbate the association between functional impairment and depressive symptoms in late life.

## Methods

### Study design and sample

A cross-sectional study design was adopted. Data for the study were drawn from the Longitudinal Ageing Study in India (LASI) wave 1 that was collected during 2017–18. It is a nationally representative survey of 72,250 individuals aged 45 years and above and their spouses (regardless of age) across all states and union territories of India. The main objective of the survey was to study the health status and the socioeconomic well-being of older adults in India [42]. The present study was conducted on eligible respondents aged 60 years and above with a sample size of 31,464 older adults (men-14,058 and women-16,366).

### Procedure

The survey adopted a three-stage sampling design in rural areas and a four-stage sampling design in urban areas. In each state/union territories (UTs), the first stage involved the selection of Primary Sampling Units (PSUs), that is, sub-districts (Tehsils/Talukas), and the second stage involved the selection of villages in rural areas and wards in urban areas in the selected PSUs. In rural areas, households were selected from selected villages in the third stage. However, sampling in urban areas involved an additional stage. Specifically, in the third stage, one Census Enumeration Block (CEB) was randomly selected in each urban area. In the fourth stage, households were selected from this CEB [42]. The goal was to select a representative sample in each stage of sample selection. Further, an individual survey schedule was administered to each consenting respondent aged 45 and above and their spouses (irrespective of age) in the sampled households. In addition, the LASI includes an individual module on biomarkers and direct health examination. The detailed methodology, with the complete information on the survey design and data collection, was published in the survey report and elsewhere [42–44]. The survey agencies that conducted the field survey for the data collection have collected prior consent from the respondents. The Indian Council of Medical Research (ICMR) extended the necessary guidelines and ethics approval for undertaking the LASI survey.

### Measures

#### Outcome variable

The outcome variable in the study is major probable depression which was coded as 0 for “not diagnosed with depression” and 1 for “diagnosed with depression”. Major depression among older adults with symptoms of dysphoria, was calculated using the Short Form Composite International Diagnostic Interview (CIDI-SF). It has 3 screening (based on the presence of dysphoria and/or anhedonia for ≥ 2 weeks during the past 12 months) and 7 symptom-based questions and a positive answer to three or more of those symptoms lead to the attribution of the label “diagnosed with depression”. The 7 symptoms are loss of interest, feeling tired, loss of appetite, trouble concentrating, feeling of worthlessness, thinking about death and trouble falling asleep [45]. This scale estimates a probable psychiatric diagnosis of major depression and has been validated in field settings and widely used in population-based health surveys [42]. The scale is a fully-structured diagnostic interview based on the Diagnostic and Statistical Manual of Mental Disorders (DSM) criteria for major depressive episode and validated in field settings especially by non-clinicians in general population surveys and in cross-cultural settings [46–48]. Cronbach’s alpha indicated that CIDI-SF has an acceptable level of reliability (α = 0.68).

#### Main explanatory variables

##### Basic Activities of Daily Living (BADL)

BADL is a term used to refer to normal daily self-care activities (such as movement in bed, changing position from sitting to standing, feeding, bathing, dressing, grooming and personal hygiene). The ability or inability to perform BADLs is used to measure a person’s functional status, especially in the case of people with disabilities and the older adults. In the LASI survey, it was assessed if a person had trouble dressing, walking across a room, eating, getting in and out of bed, washing, and using the toilet (Cronbach’s alpha = 0.87). During the interview, older individuals who struggled with any of the six activities for more than three months were labelled as facing BADL difficulty [49].

##### Instrumental activities of daily living (IADL)

These are activities that are not necessarily related to fundamental functioning of a person, but they let an individual live independently in a community. Respondents were asked if they were having any difficulties that were expected to last more than three months, such as preparing a hot meal, shopping for groceries, making a telephone call, taking medications, doing work around the house or garden, managing money (such as paying bills and keeping track of expenses), and getting around or finding an address in unfamiliar places (Cronbach’s alpha = 0.88). Those persons who had difficulty with any of the seven IADL activities for more than three months were considered facing IADL difficulty [49].

##### Marital status

It was coded as currently married and unmarried. Currently unmarried included those who were widowed/ divorced/ separated/ never married [50].

##### Living arrangements

Types of living arrangements were dichotomized into ‘co-residential living’ and ‘separate living’ [51].

##### Social participation

Following the previous studies [52, 53], survey questions based on participation in social activities were assessed to generate this variable. The activities included eating out of the house, going to park/ beach, visiting relatives/ friends, attending cultural performances/ shows/ cinema, attending religious functions/ events, and attending community/ political/ organization group meetings (Cronbach’s alpha = 0.61), and were recoded into yes and no (“yes” as 0 = at least once in a month, and “no” as 1 = rarely or never).

### Socio-demographic characteristics

Age was categorized into age groups of 60–69 years, 70–79 years, and 80 + years. Sex was coded as male and female. Educational status was coded as no education/primary, secondary and higher. Working status was coded as never worked, currently not working, working, and retired.

### Household characteristics

The monthly per-capita consumption expenditure (MPCE) quintile was assessed using household consumption data. Sets of 11 and 29 questions on the expenditures on food and non-food items (including spending on health, education, utilities, etc.), respectively, were used to canvas the sample households. Food expenditure was collected based on a reference period of seven days, and non-food expenditure was collected based on reference periods of 30 days and 365 days. Food and non-food expenditures have been standardized to the 30-day reference period [42]. The variable was divided into five quintiles, i.e., from poorest to richest. Religion was recoded as Hindu, Muslim and Others. Caste was recoded as Scheduled Caste/ Scheduled Tribe (SC/ST), Other Backward Class (OBC), and others [54]. The SC/ST are among the most disadvantaged socioeconomic groups in India. The OBC is a group of population who are intermediate in socioeconomic status, and other caste category is identified as having higher social status [55]. The place of residence was coded as urban and rural. The region was coded as North, Central, East, Northeast, West, and South.

### Statistical analyses

In this study, descriptive statistics were reported and bivariate analysis has been performed to assess the prevalence of major depression along with all key explanatory variables (BADL and IADL functioning, marital status, living arrangements and social participation). Further, moderated multivariable logistic regression models [56] were used to fulfil the objective of the study. The results are presented in the form of adjusted odds ratio (AOR) with a 95% confidence interval (CI). Individual weights were used to make the estimates nationally representative. For all the analyses, STATA version 14.2 has been used [57].

The moderated multivariable analysis provides four models to explain the adjusted estimates. Model-1 provides the estimates of depression adjusted for the control variables. Model-2, model-3, and model-4 provide the interaction effects of functional health variables (BADL and IADL functioning) with marital status, living arrangements and social participation on major depression among older adults. All the models were controlled for socio-demographic (age, gender, education and work status) and household characteristics (MPCE quintile, religion, caste, place of residence and regions).

## Results

**Fig. 2:**
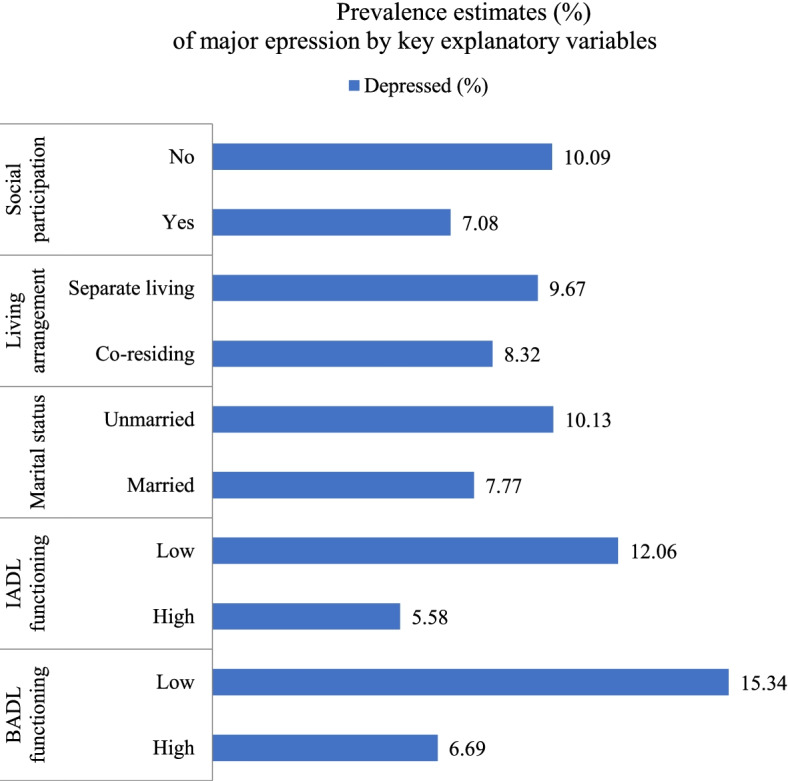
Prevalence estimates (%) of major depression by key explanatory variables

The characteristics of study sample are summarized in Table 1. More than 11% of the respondents were aged 80 + years, and 74.02% were illiterate or had primary education. The percentage of the population currently married was 61.63%. Around three-fourth of older adults were co-residing with spouse, their children or relatives. More than half of the participants were socially inactive. About one-fourth of participants reported difficulty in BADL functioning and nearly half of the respondents had difficulty in BADL functioning. Also, 8.67% of older participants were depressed. Older adults who had difficulty in BADL (15.34%), difficulty in BADL (12.06%), unmarried (10.13%), separate living (9.67%) and socially inactive (10.09) were having higher prevalence of major depression compared to their respective counterparts (Fig. 2).

**Table 1:** Participants' characteristics

Background Factors	Total (*N* = 31,464)	
	**N**	**%**
**Age (in years)**		
60–69	18,410	58.51
70–79	9,501	30.20
80 +	3,553	11.29
**Sex**		
Male	14,931	47.5
Female	16,533	52.6
**Educational status**		
No/primary	23,289	74.02
Secondary	5,741	18.24
Higher	2,434	7.74
**Working status**		
Never	8,315	26.43
Not	11,467	36.45
Yes	9,397	29.87
Retired	2,282	7.25
**Marital status**		
Currently married	19,391	61.63
Currently unmarried	12,072	38.37
**Living arrangement**		
Co-residential living	23,280	73.99
Separate living	8,184	26.01
**Social participation**		
Yes	14,525	46.83
No	16,490	53.17
**Difficulty in BADL**		
No	23,878	76.23
Yes	7,449	23.77
**Difficulty in IADL**		
No	16,162	51.64
Yes	15,133	48.36
**Depression**		
No	27,995	91.33
Yes	2,657	8.67
**MPCE quintile**		
Poorest	6,829	21.70
Poorer	6,831	21.71
Middle	6,590	20.95
Richer	6,038	19.19
Richest	5,175	16.45
**Religion**		
Hindu	25,871	82.20
Muslim	3,548	11.30
Others	2,045	6.50
**Caste**		
SC/ST	8,505	27.10
OBC	14,231	45.20
Others	8,729	27.70
**Place of residence**		
Urban	22,196	29.45
Rural	9,268	70.55
**Region**		
North	3,960	12.59
Central	6,593	20.95
East	7,439	23.64
Northeast	935	2.97
South	7,136	22.68
West	5,401	17.17

*BADL* Basic activities of daily living, *IADL* Instrumental activities of daily living, *MPCE* Monthly per capita consumption expenditure, *SC/ST* Scheduled caste/Scheduled tribe, *OBC* Other backward class

Table 2 shows the association of BADL functioning, marital status, living arrangements and social participation with major depression among older adults. The adjusted model-1 revealed that older adults who had difficulty in BADL were 2.53 times more likely to have major depression [AOR: 2.53, CI: 2.17—2.95] than those with no difficulty in BADL. The likelihood of major depression was higher among unmarried [AOR: 1.26, CI: 1.08—1.48], separate living [AOR: 1.18, CI: 1.01—1.39] and socially inactive [AOR: 1.34, CI: 1.16—1.55] older adults compared to those who were currently married, co-residing with children, spouse or relatives and socially active, respectively. When interaction term of BADL and marital status was introduced in the model-2 after controlling for all other background characteristics, the odds of having depression was higher among unmarried older adults with difficulty in BADL [AOR: 3.02, CI: 2.46—3.70] than those married with no difficulty in BADL older adults. Model 3 observed the interaction effect of BADL and living arrangements after controlling for other covariates. It was found that separate living older adults with difficulty in BADL had substantially higher likelihood [AOR: 2.98, CI: 2.32—3.83] of major depression compared to co-residing counterparts with no difficulty in BADL. Similarly, Model 4 shows that less socially active with difficulty in BADL [AOR: 3.37, CI: 2.79—4.06] had higher odds of having depression compared to socially active and functionally healthy counterparts.

**Table 2:** Association of BADL functioning, marital and living arrangement status and social participation with major depression among older adults

Variables	AOR (95% CI) Model 1	AOR (95% CI) Model 2	AOR (95% CI) Model 3	AOR (95% CI) Model 4
**Difficulty in BADL**				
No	Ref			
Yes	2.53*** (2.17—2.95)			
**Difficulty in BADL X Marital status**				
No/ Married		Ref		
No/ Unmarried		1.43*** (1.20—1.71)		
Yes/ Married		2.95*** (2.40—3.64)		
Yes/ Unmarried		3.02*** (2.46—3.70)		
**Difficulty in BADL X Living arrangement**				
No/ Co-residential living			Ref	
No/ Separate living			1.19* (0.99—1.43)	
Yes/ Co-residential living			2.55*** (2.13—3.05)	
Yes/ Separate living			2.98*** (2.32—3.83)	
**Difficulty in BADL X Social participation**				
No/ Yes				Ref
No/ No				1.46*** (1.25—1.71)
Yes/ Yes				2.93*** (2.25—3.82)
Yes/ No				3.37*** (2.79—4.06)
**Marital status**				
Married	Ref		Ref	Ref
Unmarried	1.26*** (1.08—1.48)		1.26*** (1.08—1.48)	1.26*** (1.08—1.48)
**Living arrangement**				
Co-residential living	Ref	Ref		Ref
Separate living	1.18** (1.01—1.39)	1.18** (1.01—1.39)		1.18** (1.01—1.39)
**Social participation**				
Yes	Ref	Ref	Ref	
No	1.34*** (1.16—1.55)	1.34*** (1.16—1.55)	1.34*** (1.16—1.55)	
**Age (in years)**				
60–69	Ref	Ref	Ref	Ref
70–79	0.83** (0.71—0.97)	0.83** (0.71—0.97)	0.83** (0.71—0.97)	0.83** (0.71—0.97)
80 +	0.86 (0.67—1.10)	0.87 (0.68—1.12)	0.86 (0.67—1.10)	0.87 (0.68—1.11)
**Sex**				
Male	Ref	Ref	Ref	Ref
Female	1.37*** (1.15—1.64)	1.38*** (1.16—1.65)	1.37*** (1.15—1.64)	1.37*** (1.15—1.64)
**Educational status**				
No/ primary	Ref	Ref	Ref	Ref
Secondary	0.89 (0.73—1.07)	0.89 (0.73—1.08)	0.89 (0.73—1.07)	0.89 (0.73—1.08)
Higher	0.67** (0.49—0.93)	0.68** (0.49—0.94)	0.67** (0.49—0.93)	0.68** (0.49—0.94)
**Working status**				
Never	Ref	Ref	Ref	Ref
Not	1.60*** (1.32—1.94)	1.60*** (1.32—1.94)	1.60*** (1.32—1.94)	1.59*** (1.31—1.93)
Yes	1.45*** (1.17—1.81)	1.46*** (1.18—1.82)	1.45*** (1.17—1.81)	1.45*** (1.16—1.80)
Retired	1.81*** (1.27—2.58)	1.80*** (1.26—2.58)	1.81*** (1.27—2.58)	1.79*** (1.26—2.56)
**MPCE quintile**				
Poorest	Ref	Ref	Ref	Ref
Poorer	0.93 (0.77—1.12)	0.93 (0.77—1.12)	0.93 (0.77—1.12)	0.93 (0.77—1.12)
Middle	1.02 (0.82—1.27)	1.02 (0.82—1.26)	1.02 (0.82—1.27)	1.02 (0.82—1.27)
Richer	1.17 (0.95—1.44)	1.16 (0.94—1.43)	1.17 (0.95—1.44)	1.17 (0.95—1.44)
Richest	1.40*** (1.13—1.72)	1.39*** (1.12—1.71)	1.40*** (1.13—1.72)	1.39*** (1.13—1.72)
**Religion**				
Hindu	Ref	Ref	Ref	Ref
Muslim	1.29*** (1.09—1.53)	1.29*** (1.08—1.53)	1.29*** (1.09—1.53)	1.29*** (1.09—1.53)
Others	1.09 (0.89—1.32)	1.09 (0.90—1.32)	1.09 (0.89—1.32)	1.09 (0.90—1.32)
**Caste**				
SC/ST	Ref	Ref	Ref	Ref
OBC	1.09 (0.87—1.36)	1.10 (0.88—1.37)	1.09 (0.87—1.36)	1.09 (0.87—1.36)
Others	1.20 (0.92—1.56)	1.20 (0.92—1.56)	1.20 (0.92—1.56)	1.20 (0.92—1.57)
**Place of residence**				
Urban	Ref	Ref	Ref	Ref
Rural	1.19** (1.01—1.42)	1.19** (1.01—1.42)	1.19** (1.01—1.42)	1.20** (1.01—1.42)
**Region**				
North	Ref	Ref	Ref	Ref
Central	2.21*** (1.79—2.73)	2.20*** (1.78—2.71)	2.21*** (1.79—2.73)	2.20*** (1.79—2.71)
East	1.05 (0.86—1.27)	1.04 (0.86—1.27)	1.05 (0.86—1.27)	1.05 (0.86—1.27)
Northeast	0.82 (0.61—1.11)	0.82 (0.60—1.10)	0.82 (0.61—1.11)	0.82 (0.61—1.11)
West	0.69*** (0.55—0.88)	0.69*** (0.55—0.87)	0.69*** (0.55—0.88)	0.70*** (0.55—0.88)
South	0.92 (0.73—1.17)	0.92 (0.73—1.16)	0.92 (0.73—1.17)	0.92 (0.72—1.16)
**Pseudo R2**	**0.0636**	**0.0644**	**0.0636**	**0.064**

*X* Interaction term, *BADL* Basic activities of daily living, *MPCE* Monthly per capita consumption expenditure

Table 3 shows the association of IADL functioning, marital status, living arrangements and social participation with major depression among older adults. Model 1 revealed that older adults who had difficulty in BADL were 2.27 times more likely to have major depression [AOR: 2.27, CI: 1.97—2.64] than their functionally healthy counterparts. Furthermore, older adults who belonged to older age group of 70–79 years [AOR: 0.83, CI: 0.71—0.97] and having higher education [AOR: 0.72, CI: 0.52—1.00] had a significantly lower likelihood of major depression than their counterparts. Model 2 shows the interaction effect of IADL and marital status after controlling all other background characteristics. It was noticed that unmarried older adults with difficulty in BADL had 2.8 times higher likelihood of suffering from depression than those married and having no difficulty in IADL. Similarly, model 3 observed the interaction effect of IADL difficulty and living arrangements after controlling for several socio-demoraphic variables. It was found that seperate living older adults with difficulty in IADL had a higher likelihood [AOR: 2.70, CI: 2.15—3.41] of major depression than coresiding and functionally healthy counterparts. When interaction between difficulty in IADL and social participation was analysed in Model 4, it was found that those who were less socially active with difficulty in BADL [AOR: 2.82, CI: 2.25 – 3.52] had higher odds of having depression compared to their active and functionally healthy counterparts.

**Table 3:** Association of IADL functioning and marital and living arrangement status and social participation with major depression among older adults

Variables	AOR (95% CI) Model 1	AOR (95% CI) Model 2	AOR (95% CI) Model 3	AOR (95% CI) Model 4
**Difficulty in IADL**				
No	Ref			
Yes	2.27*** (1.96—2.64)			
**Difficulty in IADL X Marital status**				
No/ Married		Ref		
No/ Unmarried		1.39*** (1.08—1.78)		
Yes/ Married		2.46*** (2.02—2.99)		
Yes/ Unmarried		2.80*** (2.22—3.52)		
**Difficulty in IADL X Living arrangement**				
No/ Co-residential living			Ref	
No/ Separate living			1.19 (0.92—1.53)	
Yes/ Co-residential living			2.27*** (1.90—2.70)	
Yes/ Separate living			2.70*** (2.15—3.41)	
**Difficulty in IADL X Social participation**				
No/ Yes				Ref
No/ No				1.06 (0.84—1.34)
Yes/ Yes				1.85*** (1.45—2.37)
Yes/ No				2.82*** (2.25—3.52)
**Marital status**				
Married	Ref		Ref	Ref
Unmarried	1.22** (1.04—1.43)		1.22** (1.04—1.43)	1.22** (1.04—1.44)
**Living arrangement**				
Co-residential living	Ref	Ref		Ref
Separate living	1.19** (1.01—1.40)	1.19** (1.01—1.40)		1.19** (1.01—1.41)
**Social participation**				
Yes	Ref	Ref	Ref	
No	1.33*** (1.14—1.54)	1.33*** (1.14—1.54)	1.33*** (1.14—1.54)	
**Age (in years)**				
60–69	Ref	Ref	Ref	Ref
70–79	0.83** (0.71—0.97)	0.83** (0.71—0.97)	0.83** (0.71—0.97)	0.83** (0.71—0.97)
80 +	0.91 (0.71—1.18)	0.92 (0.71—1.18)	0.91 (0.71—1.18)	0.90 (0.70—1.16)
**Sex**				
Male	Ref	Ref	Ref	Ref
Female	1.28*** (1.07—1.54)	1.29*** (1.07—1.55)	1.28*** (1.07—1.54)	1.28*** (1.07—1.54)
**Educational status**				
No/ primary	Ref	Ref	Ref	Ref
Secondary	0.93 (0.76—1.14)	0.93 (0.76—1.14)	0.93 (0.76—1.14)	0.93 (0.76—1.14)
Higher	0.72** (0.52—1.00)	0.73* (0.52—1.01)	0.72** (0.52—1.00)	0.70** (0.51—0.98)
**Working status**				
Never	Ref	Ref	Ref	Ref
Not	1.60*** (1.31—1.95)	1.60*** (1.31—1.95)	1.60*** (1.31—1.95)	1.60*** (1.31—1.95)
Yes	1.42*** (1.14—1.77)	1.42*** (1.14—1.77)	1.42*** (1.14—1.77)	1.42*** (1.14—1.77)
Retired	1.87*** (1.30—2.69)	1.87*** (1.30—2.68)	1.87*** (1.30—2.69)	1.87*** (1.30—2.69)
**MPCE quintile**				
Poorest	Ref	Ref	Ref	Ref
Poorer	0.91 (0.75—1.09)	0.91 (0.75—1.09)	0.91 (0.75—1.09)	0.91 (0.75—1.09)
Middle	1.05 (0.84—1.32)	1.05 (0.83—1.32)	1.05 (0.84—1.32)	1.06 (0.84—1.33)
Richer	1.16 (0.95—1.43)	1.16 (0.95—1.43)	1.16 (0.95—1.43)	1.17 (0.95—1.44)
Richest	1.42*** (1.15—1.75)	1.42*** (1.15—1.75)	1.42*** (1.15—1.76)	1.43*** (1.16—1.76)
**Religion**				
Hindu	Ref	Ref	Ref	Ref
Muslim	1.25** (1.05—1.49)	1.25** (1.05—1.49)	1.25** (1.05—1.49)	1.25** (1.05—1.49)
Others	1.08 (0.89—1.31)	1.08 (0.89—1.31)	1.08 (0.89—1.31)	1.07 (0.88—1.30)
**Caste**				
SC/ST	Ref	Ref	Ref	Ref
OBC	1.09 (0.88—1.35)	1.09 (0.88—1.36)	1.09 (0.88—1.36)	1.09 (0.88—1.35)
Others	1.22 (0.93—1.61)	1.22 (0.92—1.60)	1.22 (0.93—1.61)	1.22 (0.93—1.61)
**Place of residence**				
Urban	Ref	Ref	Ref	Ref
Rural	1.13 (0.95—1.34)	1.13 (0.95—1.34)	1.13 (0.95—1.34)	1.13 (0.95—1.34)
**Region**				
North	Ref	Ref	Ref	Ref
Central	2.37*** (1.90—2.94)	2.36*** (1.89—2.93)	2.37*** (1.90—2.94)	2.36*** (1.90—2.94)
East	1.16 (0.95—1.41)	1.15 (0.95—1.40)	1.16 (0.95—1.41)	1.15 (0.95—1.40)
Northeast	0.84 (0.62—1.14)	0.84 (0.62—1.14)	0.84 (0.62—1.14)	0.84 (0.62—1.15)
West	0.68*** (0.54—0.86)	0.68*** (0.54—0.86)	0.68*** (0.54—0.86)	0.68*** (0.54—0.86)
South	1.11 (0.88—1.41)	1.11 (0.88—1.40)	1.11 (0.88—1.41)	1.11 (0.88—1.40)
**Pseudo R2**	**0.0598**	**0.0601**	**0.0598**	**0.0607**

*X* Interaction term, *IADL* Instrumental activities of daily living, *MPCE* Monthly per capita consumption expenditure

## Discussion

The present study was set out to assess the association of low physical functioning, not being in marital union, separate living and lack of social participation with major depression among older adults in India after controlling for several socio-demographic variables. Further, the attempt was to examine the interaction effect of absence of family and lack of social participation in the association of low physical functioning with depression. The findings revealed that low physical functioning, unmarried, living alone, and less social participation were significantly associated with major depressive symptoms among the study population.

The prevalence of major depression among older adults in our study was found to be 8.9%. Rajkumar et al. found similar results in their study from rural South Indian communities [58]. On the other hand, a recent systematic review estimates pooled prevalence of depression among Indian older adults ranging from 5.5% to 80.5% [6]. Furthermore, a study using data from the Global Ageing and Adult Health (2007–2010) documented a higher prevalence (27.4%) of depression among older population in India than other low and middle-income countries such as China, Ghana, Mexico, Russia and South Africa [59]. The reason for variation might be explained by tools used to screen depression.

The current study demonstrated that difficulty in BADL and IADL functioning predicted higher amounts of depression among older adults aligning with previous research [60, 61]. The possible explanation could be that older adults with higher physical functioning are more active, resulting in relishing greater independence and more satisfying healthy lives [60]. Furthermore, deterioration of BADL and IADL is associated with loss of strength or impaired mobility that affects positive emotions of older people, which leads to vulnerability to social exclusion among older adults [61]. Owing to these circumstances, older people are unable to participate in social activities, accomplish social roles or gain identity recognition [62]. Therefore, they may experience difficulties coping with changes in their lives, resulting in deterioration in their mental health.

The results revealed that marital status was a significant predictor for major depression among older adults, indicating that currently unmarried older adults who are predominantly those who widowed in the current study have higher levels of depression than married people. This significant association is supported by substantial literature from India as well as other countries [26, 63, 64]. Gahler highlights that marital dissolution is associated with severe social discord and confers an increased risk for psychological distress and overall health deterioration [65]. Findings also indicated consistent associations between marital status and depression with physical functioning. The result of interaction effect showed that unmarried older adults with difficulty in BADL/IADL reported higher depressive symptoms. As per the marital resource model, married people have greater access to economic, psychological and social resources than unmarried individuals that can improve their physical and mental health [66]. Hence, the effect of marital status on health cannot be ignored among older adults and there is a need for future interventions with special focus on unmarried older people who are functionally impaired as they are more vulnerable to depressive symptoms.

Co-residential living can reduce the risk of depression among older adults [61, 63, 67]. This evidence has been confirmed in our study. Probably, in the joint family system, older adults have more interaction at home and the family members would be able to provide physical, social and emotional support than the case in nuclear family setup [66]. Separate living older adults with difficulty in BADL/IADL appear to have profoundly higher oods of suffering from depression in the present study. Since there are less caregivers to support older adults living in separate living arrangements where they live alone or with spouse only, functional difficulties and other health risks may create feelings of loneliness eventually leading to major depressive disorder [67]. On the other hand, older adults with higher functioning and living with family may have an adequate level of social interaction and support, resulting in better mental health as well as higher quality of life. Hence, spouse and children in co-residential living may become especially important in fulfilling the roles of intimate social contacts; therefore, support from these sources may be more effective in preventing depression that is associated with functional disability in later life.

Consistent with previous studies [61, 63, 68], social participation was a protective factor of depression. Social participation can enhance quality of life and relieve day to day life pressure that contributes lower risk of depression. Studies suggest that higher active social participation helps older adults to regulate the adverse effects of poor health condition on depressive symptoms and promote better physical and mental health in late life [69, 70]. Additionally, lack of social participation will lead to social isolation, loneliness emanating the risk of depression among older people [62, 71]. The chances of major depression was highest among older adults with difficulty in BADL/IADL and reported low social participation in our study. Enagaing in social activities may provide more physical functioning, resulting in reducing negative thoughts in daily life and improved quality of life and overall well-being of older adults [62, 72].

This study found that oldest-old respondents had lower depressive symptoms than their younger counterparts. People in older ages tend to have more experiences of negative emotions that lead to develop positive attitudes towards stressful events and increase their ability to fight health risks and have better mental health by utilizing available resources and services [73]. Concurrent with contemporary literature [6, 63], our study also indicates that older women were more prone to have major depression than men. Pilania et al. (2019) found that higher prevalence of depression among females than males (41.0% vs 28.7%) in India [6]. The possible explanation for differences between females and males may be because of biological differences as females are affected by pregnancy and related changes [74], increased vulnerability such as widowhood and living alone, especially in Indian society [6], and responsibilty of family and financial dependency on male members of the family [67].

This study found that highly educated older adults have lower chances of depression. Previous findings also reported a negative association between educational attainment and depression [58, 59, 61, 64]. Higher educational attainment can reduce the risk of depression since those who are educated may be more aware of availability and accessibility of the health services, and may have better healthcare utilization and improved health behavior among older adults [73]. Therefore, initiatives that diagnose and treat the symptoms of depression should be priortized especially among older adults with low levels of education. Interestingly, our findings showed that major depressive symptoms were reported high among older adults who belonged to the richest MPCE quintile and those individuals who were retired. Further studies are required to better understand the higher prevalenc of depression among higher economic groups which is inconsistent with existing studies [9, 59], unlike higher educational group. The higher prevalence of depression after retirement could be due to the fact that after they stop working, individuals may lose their social and psychological benefits of being active which in turn contribute to depression [67]. It is found that rural resident older adults were significantly at higher risk of experiencing depression than their urban resident counterparts and this evidence is supported by previous studies [63, 73]. It is observed that urban resident older adults have higher access of healthcare services and social resources than rural areas.

The findings of the study contribute to the broader understanding of developing intervention strategies toward the early prevention of depressive symptoms among older adults. As social support factors (marital union, co-residential living, and social participation) were found to have a significant association with deoression in older adulthood, health interventions should focus on enhancing care and support for those functionally impaired, widowed, solo living and socially inactive older individuals. Further, the use of stratified multi-stage sampling design in the country-representative LASI survey allowed the main findings of our study to be generalizable and may provide an important reference to other societies that are facing rapid population aging. Despite the aforementioned contributions, this study holds several limitations as well. First, the study is based on cross-sectional survey design. Therefore, we could not establish causal relationships among the study variables. Second, the assessment of several predictor variables in the study through self-reported information are subjected to recall and social desirability bias, resulting in exaggeration of the observed relationships. Future studies with longitudinal/cohort design are warranted on the reported associations between functional health and mental health variables and their underlying mechanisms after adjusting for several possible confounders such as healthcare utilization and other psychosocial predictors of depression which are not considered in the current study.

## Conclusion

A significant proportion of older Indian adults suffered from major depressive symptoms which calls for special attention from policymakers and health practitioners. The study suggests that low physical functioning, including its interaction with living separately, being unmarried, and lesser social participation, potentially results in major depressive symptoms among Indian older adults. The findings of the study provide insights that contribute to the development of public health intervention and highlight the importance of integrating social participation in the daily life of older adults to protect against depression associated with functional impairment. The government, as well as the community, should prioritize the programs that are based on older people's mental health through initiatives that promote a healthy surrounding such as social connectedness, co-residential living arrangements and special care for those who are physiscally disabled. Further studies using future waves of LASI should be conducted to identify other possible mechanisms of reducing the risk of depressive symptoms among older adults, especially among those who are disabled or physically ill.

## Data Availability

The study uses secondary data which is available on reasonable request through https://www.iipsindia.ac.in/content/lasi-wave-i
